# FCGR1A Serves as a Novel Biomarker and Correlates With Immune Infiltration in Four Cancer Types

**DOI:** 10.3389/fmolb.2020.581615

**Published:** 2020-12-03

**Authors:** Ji-li Xu, Yong Guo

**Affiliations:** ^1^The First Clinical Medical College, Zhejiang Chinese Medical University, Hangzhou, China; ^2^Zhejiang Provincial Hospital of Traditional Chinese Medicine, Hangzhou, China

**Keywords:** biomarker, prognosis, tumor-infiltrating, timer, GEPIA, FCGR1A

## Abstract

**Background:**

FCGR1A encodes a protein that plays an important role in the immune response. The prognostic impact and immune infiltration of FCGR1A in heterogeneous cancers remain unclear.

**Methods:**

Differential expression of FCGR1A between tumor and normal tissues and the discrepancies in overall survival (OS) among diverse cancer types were performed by Gene Expression Profiling Interactive Analysis. The correlation between FCGR1A and immune cells or gene marker sets of immune infiltrates was analyzed via Tumor Immune Estimation Resource (TIMER). Gene Ontology (GO), Kyoto Encyclopedia of Genes and Genomes (KEGG) analysis, and protein-to-protein interaction (PPI) network were used to explore the function and related genes of FCGR1A. The relationships among these genes were further analyzed by TIMER.

**Results:**

FCGR1A is highly expressed in various cancer types. FCGR1A was significantly correlated with the OS of cervical and endocervical cancer (CESC), cholangiocarcinoma (CHOL), kidney renal clear cell carcinoma (KIRC), and skin cutaneous melanoma (SKCM) (*P* < 0.05). High expression of FCGR1A meant a better prognosis besides KIRC. FCGR1A showed significant differences at different stages of KIRC and SKCM (*P* < 0.05). Furthermore, FCGR1A was notably associated with infiltrating levels of CD4^+^ T cells, CD8^+^ T cells, B cells, macrophages, neutrophils, and dendritic cells in the four cancers (*P* < 0.05). FCGR1A also showed close relevance with different immune gene markers. The copy number variation of FCGR1A significantly influenced the abundance of immune infiltration in KIRC and SKCM. GO, KEGG analysis, and PPI network analysis revealed that FCGR1A is involved in many pathophysiological processes and was most related to FCGR3A. And this gene indicated highly significant positive correlations with FCGR1A in four cancers.

**Conclusion:**

FCGR1A may be a potential prognostic biomarker and related to immune infiltration levels in diverse cancers, especially in CESC, CHOL, KIRC, and SKCM. Besides, FCGR1A may be involved in the activation, regulation, or induction of immune cells and diverse physiological and pathological processes.

## Introduction

FCGR1A (CD64) is a 72-kDa transmembrane glycoprotein with CD32 and CD16 receptors (Kiyoshi et al., 2015; Lu and Sun, 2015). It encodes a protein that plays an important role in both innate and adaptive immune response. The protein is a high-affinity Fc-gamma receptor. FCGR1A is one of three related gene family members located on chromosome 1 and often expressed on the surface of monocytes, macrophages, and dendritic cells (DCs) (Hulett and Hogarth, 1998). However, CD64 is rarely expressed on the surface of neutrophils (PMN) in normal condition. When the body is infected, CD64 expression on the surface of neutrophils can be rapidly increased under the stimulation of bacterial lipopolysaccharide, interleukin-12, interferon-γ, and granulocyte colony-stimulating factor (Mangalam and Yadav, 2019). Neutrophils are often the first immune cells to reach sites of infection, and their main function is to phagocytose and destroy invading microorganisms or foreign material. Increased CD64 expression is one of the markers of PMN activation and can be used as a good diagnostic indicator of infectious diseases (Cid et al., 2010). Previous studies have reported that FCGR1A (CD64) is of great significance for diagnosis of leukemia (Kern et al., 2006). Acute myelomonocytic leukemia (M4) and acute monocytic leukemia (M5) are both subtypes of AML, which significantly correlated with the expression of CD64 (Thepen et al., 2009).

Cervical cancer is the fourth most common cancer among women worldwide, with a high mortality rate, especially in developing countries (Bray et al., 2018). High-risk types of human papillomavirus (HPV) infections are strongly associated with a high incidence of cervical cancers; thus, HPV vaccination and screening are necessary (Crosbie et al., 2013). Immunotherapy brings hope for women with recurrent and metastatic cervical cancer (Cohen et al., 2019). Cholangiocarcinoma (CHOL) is an epithelial cell malignancy arising from diverse anatomic locations within the biliary tree, which is classified as intrahepatic, perihilar, and distal CHOL based on anatomical location (Razumilava and Gores, 2014). CHOL accounts for approximately 3% of all gastrointestinal malignancies, and the diagnosis and treatment remain extremely difficult (Khan et al., 2019). Several studies have pointed out that M2 macrophage polarization correlates with poor prognosis and metastasis of CHOL (Hasita et al., 2010; Thanee et al., 2015; Kaneda et al., 2016). Tumor-associated macrophages (TAMs) and tumor-associated neutrophil infiltration are positively associated with poor clinical outcomes, but they have not yet been considered as a therapeutic target for CHOL (Shen et al., 2014; Lee et al., 2016; Tan et al., 2016). Kidney renal clear cell carcinoma (KIRC) is the most common subtype of renal cell carcinoma (80%), and nearly 25% of patients were diagnosed at an advanced stage with lymphatic or distant metastasis (Sinha et al., 2017; Zhang B. et al., 2019). Several reports have indicated that high expression of CTLA4, LAG3, and TIGIT is related to a worse overall survival (OS), whereas high expression of TIM-3 is associated with better prognosis (Zhang S. et al., 2019). Programmed death-1 (PD-1) inhibitors (nivolumab) have been approved by the U.S. Food and Drug Administration (FDA) for the treatment of advanced RCC. CTLA-4 (ipilimumab) and PD-1 ligand inhibitors (avelumab) are currently under study (Barata and Rini, 2017). Skin cutaneous melanoma (SKCM) is one of the most aggressive cancers, accounting for 4% of all skin cancer types but 75% of skin cancer–related deaths because of its high metastatic potential (Shenenberger, 2012). The checkpoint inhibitors nivolumab (Olbryt, 2019) and pembrolizumab (Bardhan et al., 2016) have been approved by the FDA for advanced melanoma patients and have yielded a superior overall response rate (Olbryt, 2019).

In this study, we comprehensively analyzed the relationship between the expression of FCGR1A and prognosis of various cancer types using the Gene Expression Profiling Interactive Analysis (GEPIA) database. Besides, Tumor Immune Estimation Resource (TIMER) was utilized to study the correlation between FCGR1A and tumor-infiltrating immune cells. We also elaborated the important role of FCGR1A in cervical and endocervical cancer (CESC), CHOL, KIRC, and SKCM, as well as revealing a potential relationship among FCGR1A, tumor–immune interactions, and neighboring genes.

## Materials and Methods

### GEPIA Analysis

The expression level of the FCGR1A gene in distinct cancer types was analyzed via the GEPIA^1^. GEPIA is an open web that contains 9,736 tumor and 8,587 normal samples from The Cancer Genome Atlas (TCGA) and GTEx projects, which mainly including 33 cancer types (Tang et al., 2017). This web focused on the analysis of RNA sequence expression. We utilized GEPIA to plot survival curves with different FCGR1A expression in 33 cancer types, and survival differences were compared with the log-rank test. The primary endpoints of the study were OS and disease-free survival (DFS). Expression differences of FCGR1A between normal and tumor tissues were graphically visualized using box plots. Meanwhile, the effect of FCGR1A expression for diverse pathological stages was presented as violin plots.

### TIMER Database Analysis

Tumor immune estimation resource is a public resource dedicated to tumor immune infiltrates across 32 cancer types incorporating 10,897 samples from TCGA^2^ (Li et al., 2017). This web server provides a variety of immune deconvolution methods to estimate the abundance of immune infiltration and explore tumor immunity or genomic characteristic comprehensively (Li et al., 2016). Differential expression levels of FCGR1A in different cancer types were shown by the box plots via TIMER. Cancers with significant differences in FCGR1A expression would be selected for further analysis. The association between FCGR1A expression and the abundance of immune infiltrates, including CD4+ T cells, CD8+ T cells, B cell, macrophages, neutrophils, and DCs, was analyzed via gene modules. Furthermore, associations between FCGR1A and gene markers of tumor-infiltrating immune cells were also explored (Danaher et al., 2017; Siemers et al., 2017). The gene markers mainly included T cells, CD8^+^ T cells, B cells, monocyte, TAMs, neutrophils, natural killer (NK) cells, DCs, T-helper 1 (T_*H*_1) cells, regulatory T lymphocytes (Tregs), and exhausted T cells. Besides, the relationship between the copy number variation (CNV) of FCGR1A and tumor immune infiltrating level can also be analyzed easily. FCGR1A and relevant marker genes were placed on *x*- and *y*-axis, respectively, to depict scatter plots and obtain correlation coefficient as well as *P* values. The expression level was exhibited with log2 TPM.

### Related Gene Identification

Related genes with FCGR1A expression were conducted by STRING database^3^. STRING database is an online software that could perform a synthetic analysis of the direct or indirect associations among selected genes. Gene Ontology (GO) function and Kyoto Encyclopedia of Genes and Genomes (KEGG) pathway enrichment analyses of FCGR1A were performed using the STRING, and related results were obtained. The protein-to-protein interaction (PPI) networks were also constructed using the protein query function in STRING. We further analyzed the potential relationships between FCGR1A and the most related genes via TIMER database.

### Statistical Analysis

Survival curves were depicted by GEPIA. The results of GEPIA, TIMER, and STRING were displayed with hazard ratio (HR), *P* values, correlation score, or false discovery rate. The correlation of FCGR1A expression was assessed by Spearman correlation and statistical significance. In addition, the strength of the correlation should follow the following criteria: 0.00–0.19 (very weak or none), 0.20–0.39 (weak), 0.4–0.59 (moderate), 0.6–0.79 (strong), and 0.8–1.0 (very strong). *P* < 0.05 was regarded as statistically significant.

## Results

### The FCGR1A Expression Levels in Different Cancer Types

**FIGURE 1:**
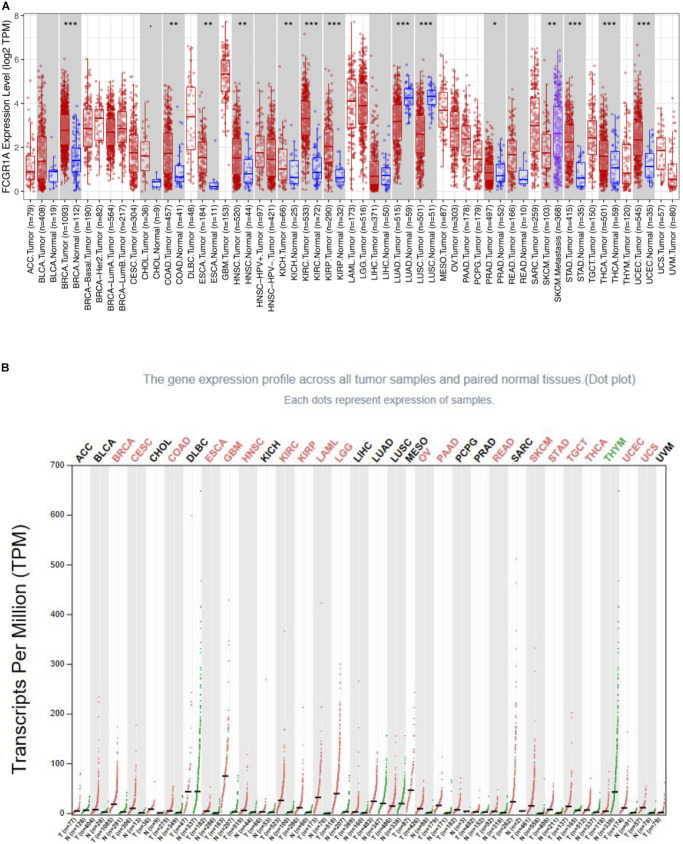
FCGR1A expression in different cancers types. **(A)** FCGR1A expression levels in different cancer types from TCGA database were performed by TIMER (*P < 0.05, **P < 0.01, ***P < 0.001). **(B)** Increased or decreased FCGR1A expression of different cancers compared with normal tissues in GEPIA.

To determine the differential expression of FCGR1A in various cancer locations, we used RNA-seq data from TCGA database to explore the relationship between prognosis and FCGR1A expression in multiple cancer types via GEPIA. Results of FCGR1A expression between tumor and normal tissues are shown in Figure 1A. To obtain more comprehensive and accurate conclusion, we also analyzed FCGR1A using the “general” module in GEPIA, and the results were exhibited in Figure 1B. Together with the above results, FCGR1A expression levels were significantly higher in bladder urothelial carcinoma (BLCA), breast invasive carcinoma (BRCA), CESC, CHOL, colon adenocarcinoma (COAD), esophageal carcinoma (ESCA), glioblastoma multiforme (GBM), head and neck cancer (HNSC), KIRC, kidney renal papillary cell carcinoma (KIRP), acute myeloid leukemia (LAML), lower-grade glioma (LGG), ovarian serous cystadenocarcinoma (OV), pancreatic adenocarcinoma (PAAD), prostate adenocarcinoma (PRAD), rectum adenocarcinoma (READ), SKCM, stomach adenocarcinoma (STAD), testicular germ cell tumor (TGCT), thyroid carcinoma (THCA), uterine corpus endometrial carcinoma (UCEC), and uterine carcinosarcoma (UCS). Conversely, FCGR1A had markedly lower expression in lung adenocarcinoma (LUAD), lung squamous cell carcinoma (LUSC), and thymoma (THYM).

### A Potential Prognostic Biomarker for CESC, CHOL, KIRC, and SKCM: FCGR1A

**FIGURE 2:**
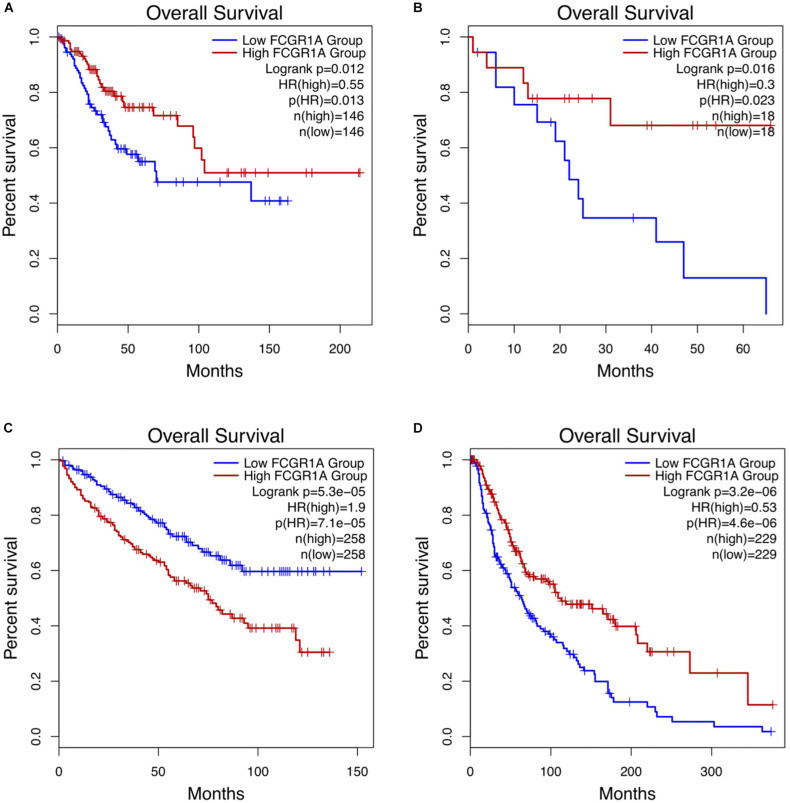
Kaplan–Meier survival curves comparing the high and low expression of FCGR1A in four cancer types in GEPIA. **(A)** CESC, **(B)** CHOL, **(C)** KIRC, and **(D)** SKCM.

**FIGURE 3:**
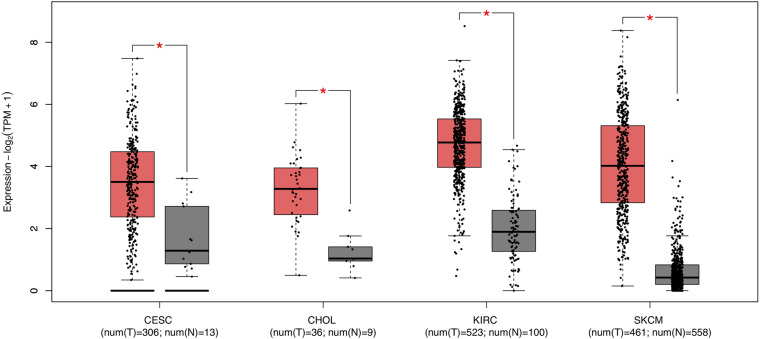
Box plots of FCGR1A expression between tumor and normal tissue in four cancer types. *P < 0.05.

**FIGURE 4:**
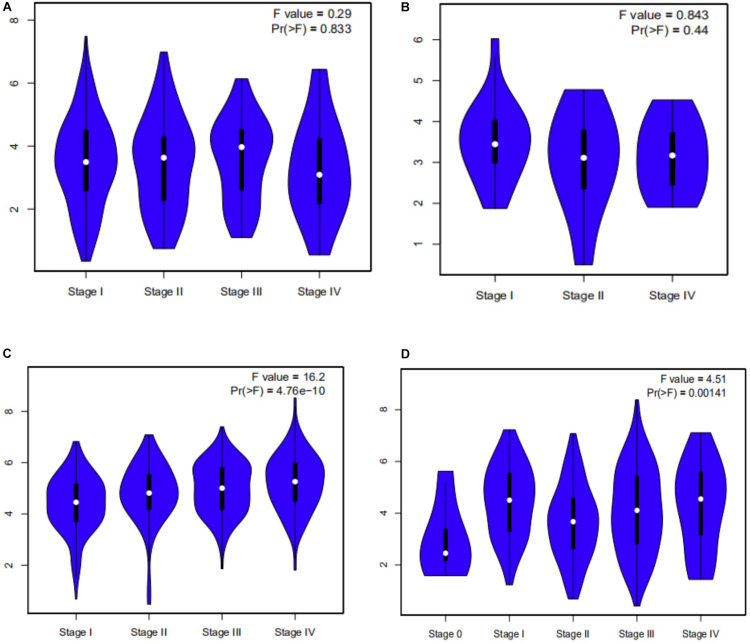
Violin plots pf FCGR1A expression in different stages of four cancers. **(A)** CESC, **(B)** CHOL, **(C)** KIRC, and **(D)** SKCM.

Survival analysis of 33 cancer types mentioned above with different FCGR1A expression was performed using GEPIA. The results showed that FCGR1A was significantly associated with prognosis in four cancer types, which were CESC, CHOL, KIRC, and SKCM, respectively (Figure 2). Based on these results, box plots indicated significant differences in FCGR1A expression levels between the tumor and normal tissues among these four cancer types (Figure 3). In addition, high FCGR1A expression exhibited superior OS in CESC (HR = 0.55, *p* = 0.012), CHOL (HR = 0.3, *p* = 0.016), and SKCM (HR = 0.53, *p* = 3.2e-06), and also showed better DFS in CHOL (HR = 0.29, *p* = 0.010). However, low expression of FCGR1A in KIRC was more beneficial to survival (HR = 1.9, *p* = 5.3e-05). FCGR1A expression levels at different stages of the four aforementioned cancer types were also analyzed. Significant differences were observed in the FCGR1A expression levels between different histological stages in KIRC (*P* = 4.76e-10) and SKCM (*P* = 0.00141). However, no significant differences were detected in CESC and CHOL (Figure 4). Thus, these results demonstrated that FCGR1A expression was linked to the prognosis of CESC, CHOL, KIRC, and SKCM. And the effect of different FCGR1A expression on four tumor locations varied.

### FCGR1A Expression and Somatic CNV Related With Immune Infiltration Level in CESC, CHOL, KIRC, and SKCM

**FIGURE 5:**
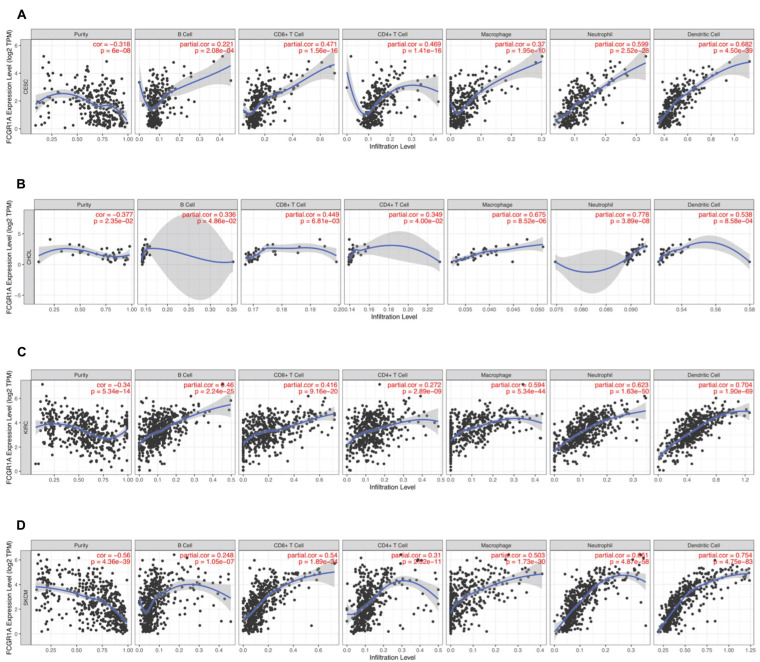
Correlation of FCGR1A expression with immune infiltration level in CESC, CHOL, KIRC, and SKCM. **(A)** CESC, **(B)** CHOL, **(C)** KIRC, and **(D)** SKCM.

To understand the correlation between FCGR1A and different infiltrated immune cells including CD4^+^ T cells, CD8^+^ T cells, B cells, macrophages, neutrophils, and DCs, TIMER was used to identify the relationship in CESC, CHOL, KIRC, and SKCM (Figure 5). All immune cells were significantly associated with FCGR1A expression levels in the four cancers (*P* < 0.05). However, relevant between FCGR1A and diverse immune cells varied substantially in different cancer types. FCGR1A had the highest correlations with DCs among CESC, KIRC, and SKCM, which were 0.682, 0.704, and 0.754, respectively. In CHOL, FCGR1A was more closely related to neutrophils (cor = 0.778). We also analyzed the correlation between FCGR1A and immune marker genes of various immune cells. The results showed that FCGR1A was notably related to most immune marker genes in CESC, CHOL, KIRC, and SKCM after purity adjustment (Table 1). And FCGR1A was strongly correlated with immune marker genes of some immune cells, which mainly concentrated in T cells, monocytes, M2 macrophages, and DCs. More specifically, immune marker genes included CD3E, CD2, CD86, CD115, CD163, VSIG4, MS4A4A, HLA-DPB1, HLA-DRA, HLA-DPA1, and ITGAX (cor ≥ 0.6). We also validated the relationship between these immune marker genes and FCGR1A expression in GEPIA and obtained similar results (Table 2). In addition, the strong correlation between FCGR1A and immune marker genes predominantly focused on CESC, KIRC, and SKCM including T cell, CD8^+^ T cell, monocyte, TAM, M2 macrophage, DC, T_*H*_1, and T cell exhaustion. Particularly, TIM-3 of the T cell exhaustion was strongly associated with CESC (cor = 0.887) and SKCM (cor = 0.934). CD86 also showed a close relation with four cancers, especially in SKCM (cor = 0.860). Nevertheless, it indicated very weak or even no correlation with four cancers in B cells, M1 macrophages, and NK cells.

**TABLE 1:** Correlation between FCGR1A and related gene markers of immune cells in TIMER.

Description	Gene markers	CESC				CHOL				KIRP				SKCM			
		None		Purity		None		Purity		None		Purity		None		Purity	
		Cor	*P*	Cor	*P*	Cor	*P*	Cor	*P*	Cor	*P*	Cor	*P*	Cor	*P*	Cor	*P*
T cell	CD3D	0.678	***	0.627	***	0.525	***	0.417	**	0.648	***	0.579	***	0.767	***	0.630	***
	CD3E	0.675	***	0.628	***	0.599	***	0.503	***	0.631	***	0.576	***	0.764	***	0.628	***
	CD2	0.710	***	0.666	***	0.596	***	0.501	***	0.668	***	0.623	***	0.806	***	0.697	***
CD8^+^ T cell	CD8A	0.672	***	0.623	***	0.542	***	0.45	***	0.613	***	0.561	***	0.813	***	0.718	***
	CD8B	0.408	***	0.348	***	0.380	***	0.287	0.094	0.596	***	0.549	***	0.769	***	0.649	***
B cell	CD19	0.408	***	0.285	***	0.391	**	0.250	0.147	0.413	***	0.364	***	0.499	***	0.320	***
	CD79A	0.436	***	0.311	***	0.374	**	0.238	0.169	0.463	***	0.412	***	0.556	***	0.369	***
Monocyte	CD86	0.813	***	0.777	***	0.772	***	0.729	***	0.847	***	0.831	***	0.906	***	0.860	***
	CD115 (CSF1R)	0.775	***	0.732	***	0.552	***	0.471	**	0.775	***	0.748	***	0.867	***	0.810	***
TAM	CCL2	0.392	***	0.302	***	0.497	***	0.454	***	0.078	0.073	0.030	0.526	0.673	***	0.556	***
	CD68	0.507	***	0.454	***	0.605	***	0.561	***	0.634	***	0.655	***	0.628	***	0.521	***
	IL10	0.525	***	0.445	***	0.411	**	0.230	0.184	0.646	***	0.596	***	0.720	***	0.622	***
M1 Macrophage	IRF5	0.199	***	0.193	***	0.291	0.086	0.291	0.200	0.385	***	0.385	***	0.650	***	0.493	***
	INOS (NOS2)	−0.127	**	−0.164	***	−0.228	0.180	−0.249	0.148	−0.037	0.400	−0.106	**	0	0.993	−0.011	0.822
	COX2 (PTGS2)	−0.228	***	−0.342	***	0.387	**	0.287	0.095	0.131	***	0.089	0.055	0.046	0.318	−0.037	0.428
M2 macrophage	CD163	0.827	***	0.801	***	0.669	***	0.600	***	0.682	***	0.673	***	0.803	***	0.743	***
	VSIG4	0.794	***	0.772	***	0.783	***	0.742	***	0.782	***	0.772	***	0.859	***	0.830	***
	MS4A4A	0.858	***	0.836	***	0.733	***	0.686	***	0.738	***	0.714	***	0.834	***	0.773	***
Neutrophils	CD11b (ITGAM)	0.606	***	0.539	***	0.425	**	0.370	**	0.711	***	0.702	***	0.772	***	0.706	***
	CCR7	0.410	***	0.316	***	0.516	**	0.395	***	0.490	***	0.440	***	0.599	***	0.371	***
Natural killer cell	KIR2DL1	0.319	***	0.239	***	−0.043	0.802	−0.107	0.539	0.036	0.405	−0.006	0.904	0.380	***	0.237	***
	KIR2DL3	0.465	***	0.401	***	0.05	0.771	0.006	0.971	0.058	0.178	0.047	0.315	0.561	***	0.402	***
	KIR2DL4	0.390	***	0.340	***	0.236	0.165	0.168	0.334	0.279	***	0.247	***	0.711	***	0.590	***
	KIR3DL1	0.400	***	0.303	***	0.210	0.219	0.170	0.330	0.040	0.361	0.052	0.262	0.503	***	0.347	***
	KIR3DL2	0.497	***	0.435	***	0.331	**	0.346	**	0.108	**	0.088	0.059	0.108	***	0.393	***
	KIR3DL3	0.323	***	0.226	***	−0.189	0.270	−0.262	0.128	0.084	0.051	0.057	0.262	0.174	***	0.112	**
	KIR2DS4	0.371	***	0.318	***	0.116	0.499	0.066	0.708	0.041	0.341	0.018	0.693	0.441	***	0.313	***
Dendritic cell	HLA-DPB1	0.628	***	0.580	***	0.644	***	0.573	***	0.755	***	0.732	***	0.822	***	0.728	***
	HLA-DQB1	0.502	***	0.447	***	0.486	**	0.425	0.088	0.442	***	0.394	***	0.758	***	0.637	***
	HLA-DRA	0.570	***	0.524	***	0.654	***	0.581	***	0.775	***	0.764	***	0.843	***	0.760	***
	HLA-DPA1	0.597	***	0.549	***	0.696	***	0.634	***	0.736	***	0.710	***	0.813	***	0.725	***
	BDCA-1 (CD1C)	0.176	***	0.113	0.061	0.417	**	0.300	0.080	0.241	***	0.162	***	0.459	***	0.232	***
	BDCA-4 (NRP1)	0.229	***	0.176	***	0.587	***	0.529	**	0.059	0.173	0.003	0.954	0.432	***	0.363	***
	CD11c (ITGAX)	0.672	***	0.617	***	0.587	***	0.496	***	0.572	***	0.561	***	0.648	***	0.503	***
Helper T cell 1 (T_*H*_1)	T-bet (TBX21)	0.721	***	0.684	***	0.562	***	0.453	***	0.297	***	0.245	***	0.792	***	0.677	***
	STAT4	0.491	***	0.388	***	0.007	0.970	−0.123	0.482	0.410	***	0.348	***	0.666	***	0.511	***
	STAT1	0.562	***	0.512	***	0.245	0.149	0.198	0.253	0.614	***	0.579	***	0.687	***	0.619	***
	IFNG	0.699	***	0.644	***	0.440	***	0.322	0.059	0.587	***	0.545	***	0.783	***	0.683	***
	TNF	0.106	0.064	0.042	0.482	0.374	**	0.337	**	0.402	***	0.378	***	0.642	***	0.473	***
Regulatory cell (Treg)	FOXP3	0.583	***	0.517	***	0.543	***	0.437	***	0.577	***	0.532	***	0.629	***	0.438	***
	CCR8	0.494	***	0.434	***	0.505	**	0.416	**	0.564	***	0.525	***	0.669	***	0.533	***
	TGFB1 (TGFβ)	0.161	***	0.080	0.184	0.387	**	0.314	0.067	0.222	***	0.160	***	0.456	***	0.325	***
T cell exhaustion	PD-1 (PDCD1)	0.715	***	0.665	***	0.376	**	0.306	0.074	0.586	***	0.547	***	0.775	***	0.657	***
	CTLA4	0.684	***	0.625	***	0.426	**	0.345	**	0.516	***	0.467	***	0.498	***	0.323	***
	LAG3	0.734	***	0.689	***	0.364	**	0.26	0.131	0.614	***	0.573	***	0.819	***	0.732	***
	TIM-3 (HAVCR2)	0.887	***	0.867	***	0.677	***	0.612	***	0.294	***	0.243	***	0.934	***	0.901	***
	GZMB	0.607	***	0.537	***	0.484	**	0.382	***	0.291	***	0.214	***	0.759	***	0.622	***

***P < 0.01, **P < 0.05.

**TABLE 2:** Correlation between FCGR1A and related gene markers of immune cells in GEPIA.

Description	Gene markers	CESC	CHOL	KIRP	SKCM
		**Cor *P***	**Cor *P***	**Cor *P***	**Cor *P***
T cell	CD3D	0.627 ***	0.417 ***	0.597 ***	0.63 ***
	CD3E	0.628 ***	0.503 ***	0.576 ***	0.628 ***
	CD2	0.666 ***	0.501 ***	0.623 ***	0.697 ***
CD8^+^ T cell	CD8A	0.623 ***	0.45 ***	0.561 ***	0.718 ***
	CD8B	0.348 ***	0.287 ***	0.549 ***	0.649 ***
Monocyte	CD86	0.770 ***	0.750 ***	0.760 ***	0.860 ***
	CD115 (CSF1R)	0.690 ***	0.63 ***	0.69 ***	0.800 ***
Macrophage	IRF5	0.12 0.039	0.58 ***	0.27 ***	0.62 ***
	CD163	0.840 ***	0.24 0.110	0.700 ***	0.800 ***
	VSIG4	0.740 ***	0.742 ***	0.700 ***	0.830 ***
	MS4A4A	0.810 ***	0.570 ***	0.660 ***	0.780 ***
Dendritic cell (DC)	HLA-DPB1	0.580 ***	0.730 ***	0.710 ***	0.790 ***
	HLA-DQB1	0.440 ***	0.440 **	0.510 ***	0.650 ***
	HLA-DRA	0.530 ***	0.700 ***	0.700 ***	0.820 ***
	HLA-DPA1	0.540 ***	0.700 ***	0.670 ***	0.770 ***
	BDCA-1 (CD1C)	0.120 **	0.410 ***	0.120 ***	0.430 ***
	BDCA-4 (NRP1)	0.140 **	0.660 ***	0.130 ***	0.340 ***
	CD11c (ITGAX)	0.550 ***	0.650 ***	0.600 ***	0.610 ***
Helper T cell 1 (T_*H*_1)	T-bet (TBX21)	0.680 ***	0.370 **	0.480 ***	0.780 ***
	STAT4	0.460 ***	0.260 0.083	0.540 ***	0.650 ***
	STAT1	0.490 ***	0.520 ***	0.420 ***	0.640 ***
	IFNG	0.690 ***	0.420 ***	0.690 ***	0.790 ***
	TNF	0.098 0.084	0.530 **	0.290 ***	0.610 ***
T cell exhaustion	PD-1 (PDCD1)	0.700 ***	0.490 ***	0.680 ***	0.770 ***
	CTLA4	0.680 ***	0.560 ***	0.630 ***	0.480 ***
	LAG3	0.730 ***	0.150 0.31	0.720 ***	0.820 ***
	TIM-3 (HAVCR2)	0.840 ***	0.760 ***	0.350 ***	0.900 ***
	GZMB	0.640 ***	0.560 **	0.520 ***	0.780 ***
	CTLA4	0.680 ***	0.560 ***	0.630 ***	0.480 ***

***P < 0.01, **P < 0.05.

**FIGURE 6:**
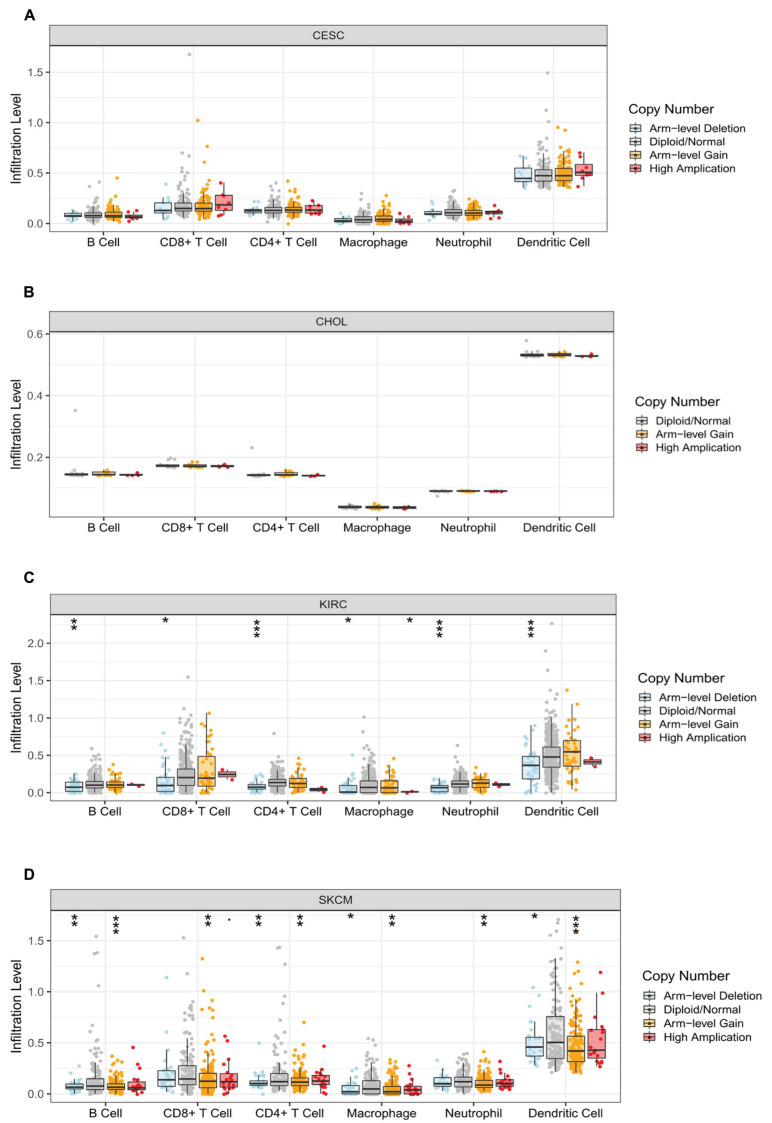
Correlation between somatic copy number variation and immune infiltration levels of six immune cells in four cancers. **(A)** CESC, **(B)** CHOL, **(C)** KIRC, and **(D)** SKCM (*P < 0.05, **P < 0.01, ***P < 0.001).

Relevance between somatic CNV and immune infiltration using the SNCA module in TIMER differed among four cancers. Studies showed that arm-level deletion in KIRC was significantly associated with the infiltration of B cells, CD8^+^ T cells, CD4^+^ T cells, macrophages, neutrophils, and DCs (*P* < 0.05). Moreover, high amplification was related to the level of macrophage infiltration (*P* < 0.05). In SKCM, arm-level gain was prominently correlated with six immune cells, whereas arm-level deletion was related only to B cells, CD4^+^ T cells, macrophages, and DCs (*P* < 0.05). No association was observed between CNV and immune cell infiltration in the other two cancer types (*P* > 0.05) (Figure 6).

### Function Analysis of the PPI Network

**FIGURE 7:**
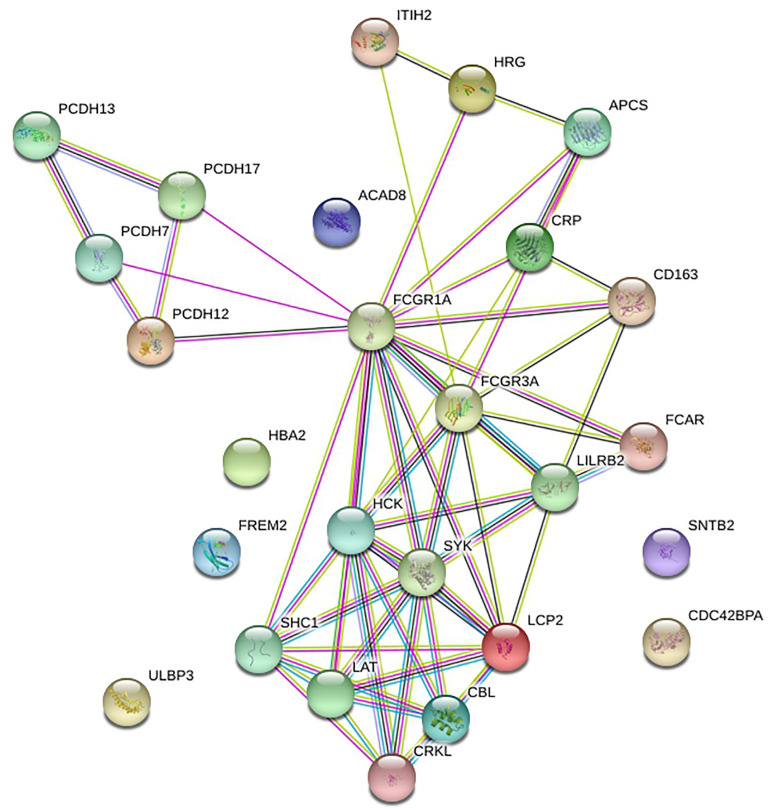
Protein–protein interaction network of FCGR1A.

STRING was utilized to construct the PPI network, and GO and KEGG analyses were performed to identify the enrichment of FCGR1A in GO terms and metabolic pathways (Table 3). The GO enrichment analysis consisted of three parts: GO biological process (GO-BP), GO molecular function (GO-MF), and GO cellular component (GO-CC). GO-BP included 115 GO-terms, the most important of which were Fc receptor signaling pathway and immune response–regulating cell surface receptor signaling pathway. GO-MF and GO-CC contained 17 and 21 GO terms, respectively. GO-BF contained 17 items, and FCGR1A was largely involved in phosphotyrosine residue binding, immunoglobulin binding, and calcium ion binding. Meanwhile, FCGR1A was enriched for 21 GO-CC terms, and plasma membrane, whole membrane, and cytoplasmic vesicle were the top three categories. Eighteen items were included in the KEGG pathways analysis. The results indicated that FCGR1A was predominantly associated with Fc gamma R–mediated phagocytosis, osteoclast differentiation, and NK cell–mediated cytotoxicity. The PPI network displayed intricate relationship between FCGR1A and other genes in Figure 7. FCGR3A, SYK, and HCK were top three significant genes related to the FCGR1A according to the correlation score (Table 4). The relationship between FCGR3A and FCGR1A displayed highly significant positive correlation in four cancers (Supplementary Figure 1).

**TABLE 3:** Enriched GO and KEGG items.

Category	Description	Count in network	False discovery rate
GO-BP	Fc receptor signaling pathway	8 of 126	6.36E-09
	Immune response–regulating cell surface receptor Signaling pathway	9 of 266	2.74E-08
	Regulation of immune response	11 of 873	1.79E-06
	Defense response	12 of 1,234	4.06E-06
	Immune system process	15 of 2,370	7.35E-06
GO-MF	Phosphotyrosine residue binding	5 of 37	3.40E-07
	Immunoglobulin binding	4 of 21	1.52E-06
	Calcium ion binding	7 of 700	0.00078
	Protein-containing complex binding	8 of 968	0.00078
	Complement component C1q binding	2 of 8	0.0016
GO-CC	Plasma membrane	19 of 5,159	0.00013
	Whole membrane	10 of 1,554	0.0008
	Cytoplasmic vesicle	11 of 2,226	0.0023
	Cytoplasmic vesicle membrane	6 of 724	0.006
	Plasma membrane raft	3 of 95	0.006
KEGG pathway	Fc gamma R–mediated phagocytosis	5 of 89	7.07E-06
	Osteoclast differentiation	5 of 124	1.72E-05
	Natural killer cell–mediated cytotoxicity	5 of 124	1.72E-05
	*Staphylococcus aureus* infection	3 of 51	0.00063
	Bacterial invasion of epithelial cells	3 of 72	0.0013

*BP, biological process; MF, molecular function; CC, cellular component.*

**TABLE 4:** The correlation score between FCGR1A and neighboring genes.

Node1	Node2	Correlation score
FCGR1A	FCGR3A	0.977
	SYK	0.976
	HCK	0.974
	LILRB2	0.921
	CD163	0.851
	CRP	0.844
	PCDH17	0.823
	PCDH7	0.795
	FCAR	0.792
	HRG	0.663
	PCDH12	0.570
	LCP2	0.537
	APCS	0.492
	LAT	0.418
	SHC1	0.412

## Discussion

FCGR1A (CD64) is the single high-affinity receptor of immunoglobulin G (IgG) in human. The expression of FCGR1A is closely related to immune inflammation. Although there are few extensive and systematical studies about CD64, its high sensitivity and specificity for the diagnosis of sepsis and systemic infection had been demonstrated, with a sensitivity of greater than 90% and specificity of 90–100% in adults and children (Hoffmann, 2009). Besides, elevated CD64 had been reported as a potential biomarker for the diagnosis of PFAPA (periodic fever, aphthous stomatitis, pharyngitis, and adenitis) syndrome (Yamazaki et al., 2014). FCGR1A is the encoding gene of CD64, and the up-regulation of FCGR1A leads to high expression of CD64. CD64 plays an important role in resistance to pathogen invasion through antibody-dependent cell-mediated cytotoxicity (ADCC), cell phagocytosis, and clearance of immune complexes. In normal conditions, CD64 is mainly expressed in monocytes, macrophages, and DCs but lower expression in neutrophils and lymphocytes. When the body or cells are stimulated by endotoxin or immune factors, the expression level of CD64 in the neutrophils increases, and FCGR1A is up-regulated. Here, we studied the association between FCGR1A and the prognosis of different cancers. High expression of FCGR1A exhibited a superior survival in CESC, CHOL, and SKCM, but associated with a poor prognosis in KIRC. Moreover, our research showed that immune infiltration levels and various immune marker genes were closely related to the FCGR1A expression levels. Thus, we can infer that FCGR1A can be regarded as a potential biomarker of cancers and play a role in tumor immunology.

We explored the expression levels of FCGR1A in 33 cancer types using TCGA data in GEPIA. The significant differential expression of FCGR1A was observed in many cancer types. In contrast to the normal tissue, FCGR1A expressed highly in BLCA, BRCA, CESC, CHOL, COAD, ESCA, GBM, HNSC, KIRC, KIRP, LAML LGG, OV, PAAD, PRAD, READ, SKCM, STAD, TGCT, THCA, UCEC, and UCS, but lowly in LUAD, LUSC, and THYM. Moreover, FCGR1A expression levels were significantly correlated with the OS of CESC, CHOL, KIRC, and SKCM and had notable differences between tumor and normal tissues in these four cancers. Besides, FCGR1A expression in KIRC and SKCM showed conspicuous discrepancies at different stages. Taken together with the above results, FCGR1A was strongly confirmed as a prognostic biomarker in CESC, CHOL, KIRC, and SKCM.

This study also indicated that FCGR1A was associated with multiple levels of immune infiltration in cancers. FCGR1A had the strongest correlation with DCs among six tumor-infiltrating immune cells in CESC, KIRC, and SKCM. It had higher relevancy with neutrophils in CHOL as well. Furthermore, significant positive relevance between FCGR1A expression and six immune cells was discovered in four cancers. CD3E, CD2, CD86, CD163, VSIG4, MS4A4A, HLA-DPB1, HLA-DRA, HLA-DPA1, and TIM-3 were found to be mainly gathering in T cells, monocytes, macrophages, and DCs were significantly correlated with FCGR1A. Similar results were validated in GEPIA. Therefore, the association of FCGR1A with diverse immune cell marker genes suggested its role in regulating tumor immunology in CESC, CHOL, KIRC, and SKCM. M1 macrophage gene markers (such as NOS2 and IRF5) had weak or no correlation with FCGR1A expression, while M2 macrophage markers CD163, VSIG4, and MS4A4A had strong correlations. These consequences indicated the potential regulatory role of FCGR1A in the polarization of TAMs. The increased expression of FCGR1A was positively correlated with the expression of Tregs and T cell exhaustion markers (FOXP3, CCR8, PD-1, Tim-3, and LAG3), suggesting that FCGR1A may play a role in the activation of Tregs and induction of T cell exhaustion. The FCGR1A was moderately or strongly associated with most immune marker genes in DCs, which can induce immune memory responses in cancer and promote anti-tumor immunity. Thus, the relationship between FCGR1A expression and the infiltration level of DCs may predict the prognosis of the disease and the response to therapies. TIM-3 is a vital surface protein on T cell exhaustion (Huang et al., 2015) and is highly correlated with FCGR1A expression in CESC and SKCM. Literature had reported that patients with high expression of TIM-3 had high metastatic potential, advanced cancer grades, and shorter OS than those with low expression in cervical cancer (Cao et al., 2016). And some studies had pointed out that TIM-3 is an immunomodulatory molecule in melanoma cells (Wiener et al., 2007). Besides, FCGR1A was positively correlated with T helper cells (such as T_*H*_1) and may be involved in the regulatory role of T cells. Taken together, FCGR1A plays a crucial role in the activation and regulation of immune cells in CESC, CHOL, KIRC, and SKCM based on the above evidence.

To have a better understanding of FCGR1A, GO enrichment and KEGG pathway analysis was carried out. FCGR1A was involved in various biological process, molecular function, and cellular component, which mainly includes Fc receptor signaling pathway, immune response–regulating cell surface receptor signaling pathway, phosphotyrosine residue binding, immunoglobulin binding, plasma membrane, whole membrane, etc. KEGG analysis was used to identify significant signaling pathways. Among its related pathways, Fc gamma R–mediated phagocytosis and osteoclast differentiation were the most important. According to the PPI network and correlation score, FCGR3A, SYK, and HCK were the most relevant neighboring genes. FCGR3A (low-affinity immunoglobulin gamma Fc region receptor III-A) was the receptor for the Fc region of IgG and bound complexed or aggregated IgG and monomeric IgG. It mainly mediated ADCC and other antibody-dependent responses (Mahaweni et al., 2018). SYK was a non-receptor tyrosine kinase, which mediates signal transduction downstream of transmembrane receptors including classical immunoreceptors (Mócsai et al., 2010). HCK was found in hematopoietic cells that transmitted signal from cell surface receptors and regulated innate immune responses. It also acted downstream of receptors that bound the Fc region of immunoglobulins, such as FCGR1A, FCGR2A, CSF3R, etc. (Yang et al., 2016). These three genes were all demonstrated to be associated with various cancer types or drug responses (Fueyo et al., 2018; Magnes et al., 2018; Roseweir et al., 2019). Our findings also confirmed that FCGR3A had highly significant positive associations with FCGR1A in four cancers. This evidence may help us infer the correlation between FCGR1A and cancers. In addition, we sought to investigate the relationship between FCGR1A and some genes that were confirmed to be related to cancers. Here, we took CASS4 (HEPL) as an example. CASS4 (Cas scaffold protein family member 4) was a protein coding gene and played a role in tyrosine kinase-base signaling related to cell adhesion and cell spreading, as well as regulating PTK2/FAK1 activity and focal adhesion integrity (Singh et al., 2008). The published research showed that CASS4 overexpression increased cell spreading and FAK activation. And it may be most in connection with the normal function of lung and hematopoietic system. We explored the correlation between FCGR1A and CASS4 in various cancer types through TIMER database. The results indicated that FCGR1A and CASS4 showed positive correlations in all cancer types including the four cancers we studied (*P* < 0.05, ρ > 0) (Supplementary Figure 2). Meanwhile, genemania^4^ was used to construct the PPI network (Supplementary Figure 3). Although there was no direct correlation between CASS4 and FCGR1A, these two genes were both associated with CD68, CCR1, CD300C, LILRA5, and PLEKHS1. The underlying mechanisms for gene-specific interplay among these two genes may be worthy of further investigation.

Several studies on FCGR1A (CD64) mainly focused on the diagnosis and distinction of infectious diseases, but rarely on cancer. However, an experimental study published in 2013 pointed out that FcRI (CD64) could mediate the protective effect of anti-tumor antibodies on melanoma metastasis and may also have a protective effect on solid tumors (Mancardi et al., 2013). In addition, CD64 is often used in combination with C-reactive protein (CRP), procalcitonin (PCT), and other inflammatory markers for diagnosis (Yeh et al., 2019). Multiple studies have demonstrated the predictive value of CRP in cancer outcomes. They showed that CRP was generally 1.3-fold higher than normal level in all cancer types. In lung cancer, CRP displayed more than 2-fold higher than normal value and increased approximately 80% risk of early mortality (Allin and Nordestgaard, 2011). Moreover, elevated CRP at diagnosis was associated with poor prognosis in breast cancer (Allin et al., 2011). A study proposed that PCT was closely related to the diagnosis and outcome of lung cancer (Pardo-Cabello and Manzano-Gamero, 2019). Therefore, infectious indicators are also of great value in cancer diagnosis and prognosis prediction and might be a novel promising biomarker. However, at present, insufficient information is available about FCGR1A; further experimental and clinical studies should be conducted to validate our findings.

## Conclusion

In summary, high FCGR1A expression leads to different outcomes in diverse cancers. It is associated with better prognosis in CESC, CHOL, and SKCM besides CHOL. Meanwhile, FCGR1A is positively or even strongly correlated with immune cells and various immune cell marker genes in four cancer types, such as CD2, CD3E, CD86, CD163, VSIG4, MS4A4A, HLA-DPB1, HLA-DRA, HLA-DPA1, ITGAX, and TIM-3 in T cell, monocyte, M2 macrophage, DC, T_*H*_1, and T cell exhaustion. Thus, FCGR1A may contribute to the activation and regulation of the aforementioned immune cells to varying degrees. FCGR1A may play an important role in immune cell infiltration and serve as a prognostic biomarker for four cancers, especially in SKCM. Also, FCGR1A is involved in many pathophysiological processes and is closely related to FCGR3A, SYK, and HCK in various cancers. It seems that many questions need to be addressed by future research.

## Data Availability Statement

Publicly available datasets were analyzed in this study. This data can be found here: http://gepia2.cancer-pku.cn/#general and https://cistrome.shinyapps.io/timer/.

## Author Contributions

J-lX: conceptualization, formal analysis and investigation, and writing-original draft preparation. YG: conceptualization, methodology, writing-review and editing, and supervision. Both authors contributed to the article and approved the submitted version.

## Conflict of Interest

The authors declare that the research was conducted in the absence of any commercial or financial relationships that could be construed as a potential conflict of interest.

## Footnotes

1 http://gepia.cancer-pku.cn/index.html

2 https://cistrome.shinyapps.io/timer/

3 https://string-db.org/

4 http://genemania.org
