# Supplementary material for: FCGR1A Serves as a Novel Biomarker and Correlates With Immune Infiltration in Four Cancer Types
Source: Front Mol Biosci. 2020 Dec 3;7:581615. doi: 10.3389/fmolb.2020.581615 (PMC7744780; doi:10.3389/fmolb.2020.581615)

Supplementary Figure 1 The correlation between FCGR1A and FCGR3A in CESC, CHOL, KIRC and SKCM

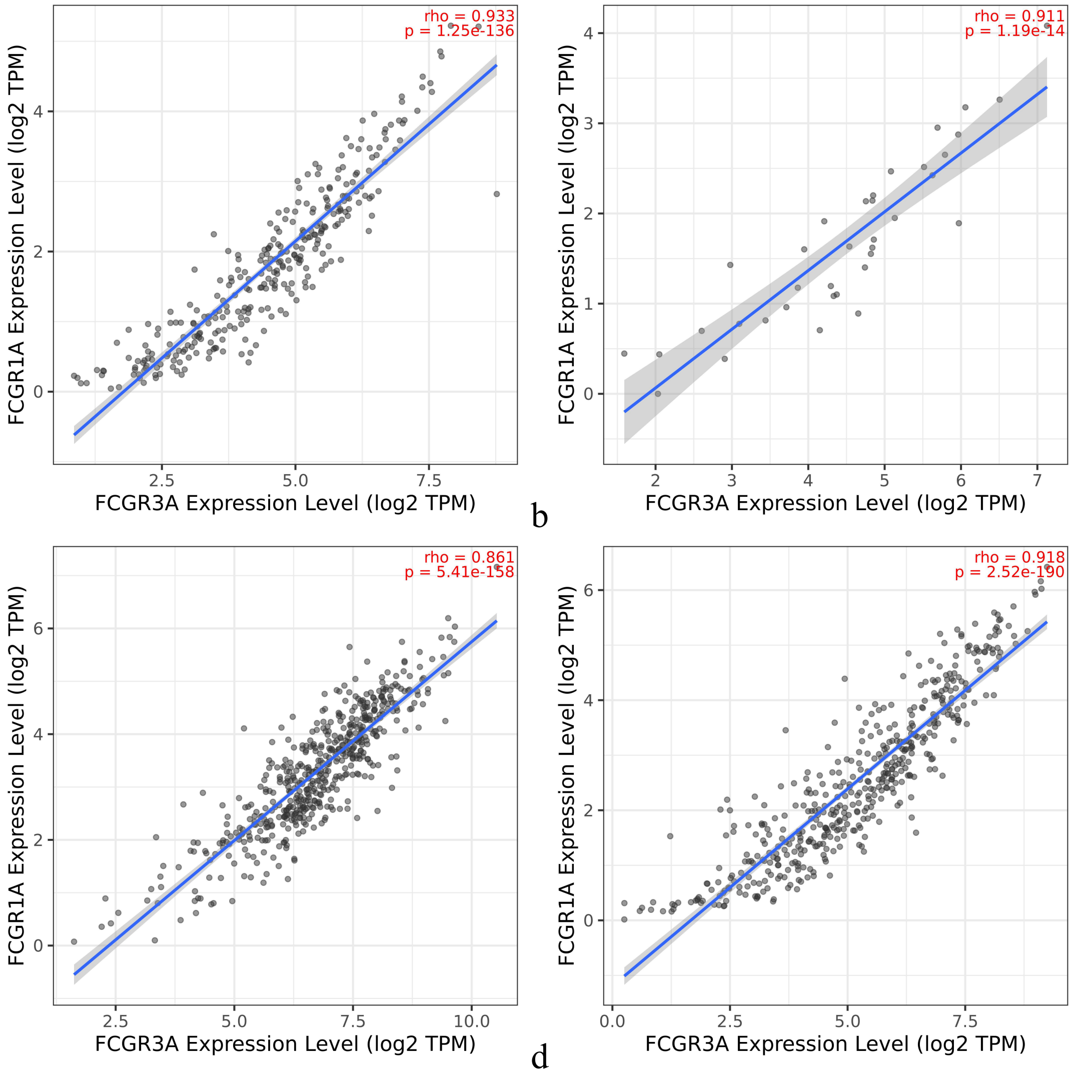

a: CESC; b: CHOL; c:KIRC; d: SKCM

Supplementary Figure 2 The correlation between FCGR1A and CASS4 in CESC, CHOL, KIRC and SKCM

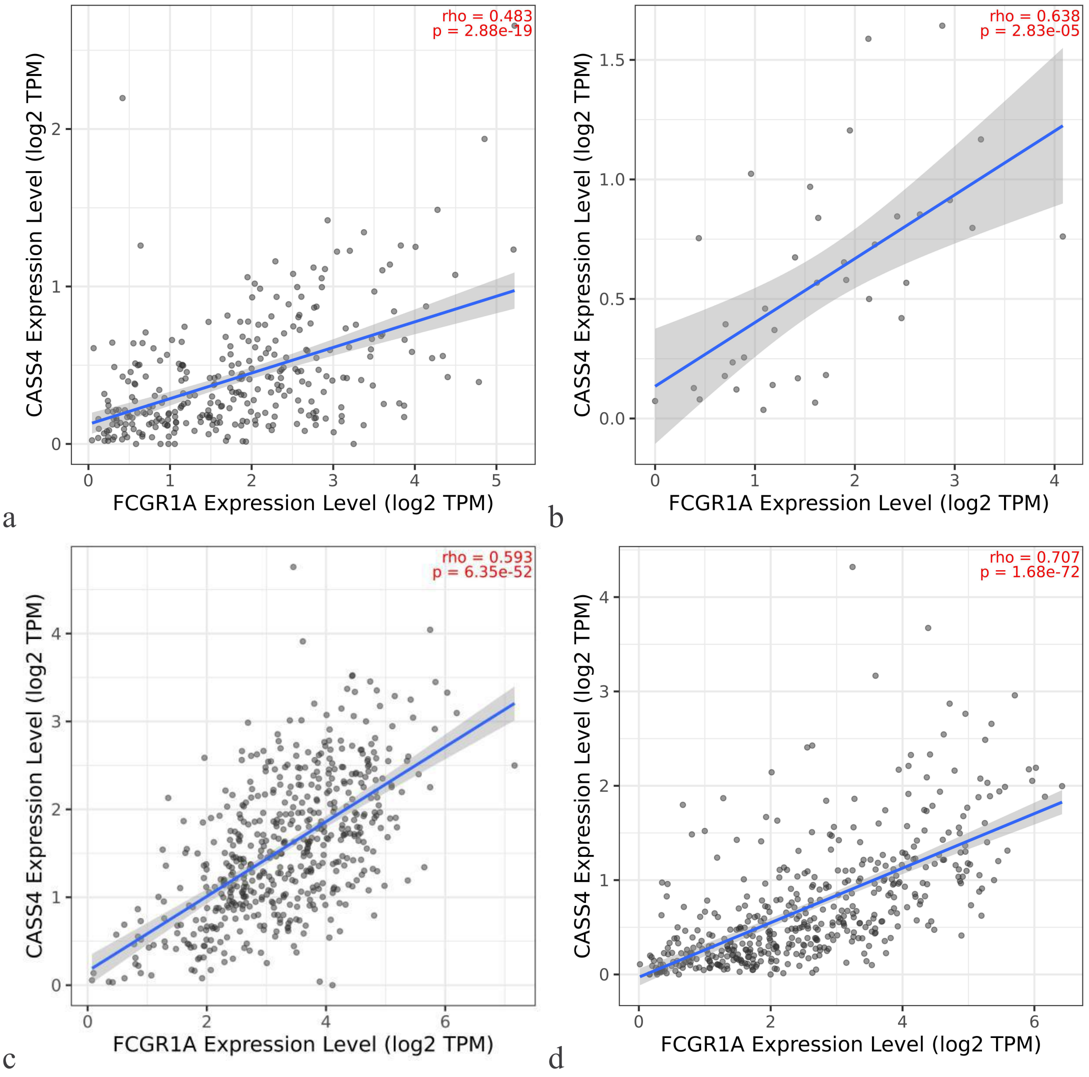

a: CESC; b: CHOL; c:KIRC; d: SKCM

Supplementary Figure 3 PPI network between FCGR1A and CASS4

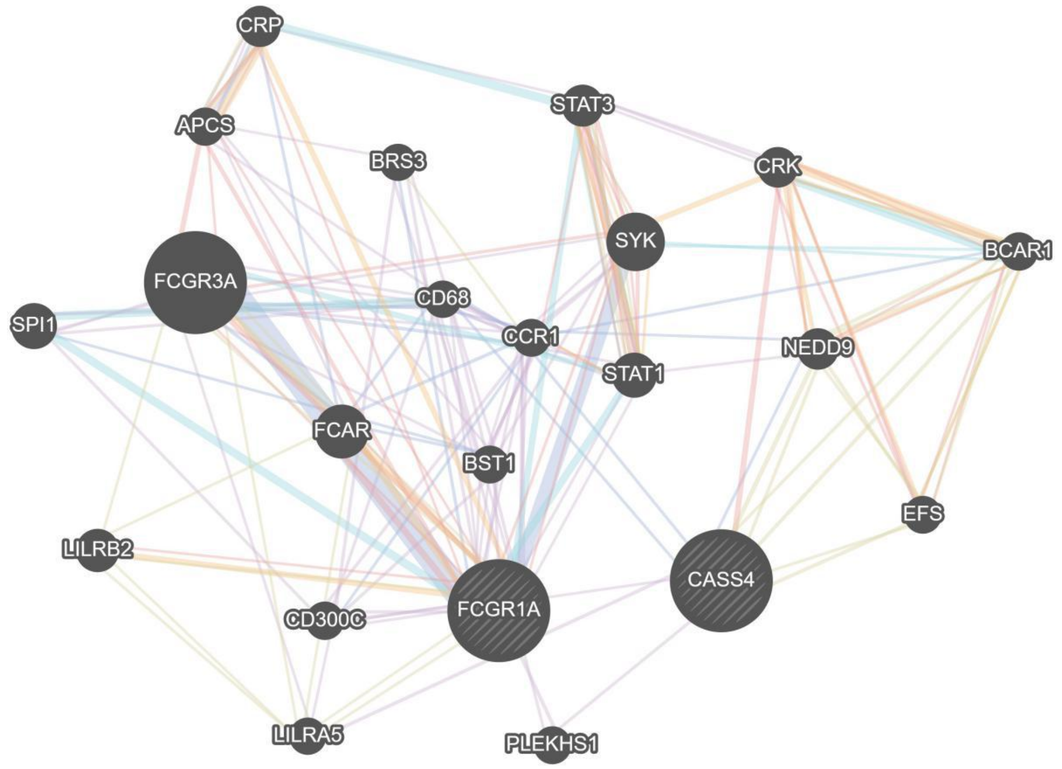

Supplement: Supplementary file 1 [file Data_Sheet_1.PDF]
